# Transition of the insertion of nurse midwives in the delivery room of a teaching hospital

**DOI:** 10.1590/0034-7167-2025-0190

**Published:** 2026-10-19

**Authors:** Thalita Rocha Oliveira, Valdecyr Herdy Alves

**Affiliations:** I Instituto Nacional de Saúde da Mulher, da Criança e do Adolescente Fernandes Figueira/Fiocruz. Rio de Janeiro, Rio de Janeiro. Brazil; II Universidade Federal Fluminense. Niterói, Rio de Janeiro, Brazil

**Keywords:** Nurse Midwives, Hospitals, Teaching, Delivery Rooms, Maternal-Child Health Services, Health Policy., Enfermeras Obstetrices, Hospitales de Enseñanza, Salas de Parto, Servicios de Salud Materno-Infantil, Política de Salud.

## Abstract

**Objectives:**

to understand the transition of the insertion of nurse midwives in the delivery room based on the Apice On Project in a teaching hospital.

**Methods:**

a mixed-method study - Embedded Concurrent Design. Qualitative data were collected from 18 professionals from a maternity hospital in the city of Rio de Janeiro through semi-structured interviews and submitted to content analysis. Quantitative data were extracted from the obstetric indicators logbook and processed using descriptive statistical analysis.

**Results:**

the correlation of data indicated determining factors for the performance of specialists: institutional profile, deficiencies in communication flows regarding the Apice On guidelines, and limitations of residency programs in articulating principles of interprofessional education for collaborative practice in obstetrics.

**Final Considerations:**

experiencing processes of organizational transition in care models requires horizontal communication, awareness, professional engagement, and role definition to overcome institutional barriers.

## RESUMEN

**Objetivos:**

comprender la transición hacia la incorporación de enfermeras obstétricas en las salas de parto a partir del Proyecto Apice On en un hospital universitario.

**Métodos:**

estudio de método mixto: proyecto incorporado concomitante. Los datos cualitativos se recopilaron de 18 profesionales de una maternidad del municipio de Río de Janeiro mediante entrevistas semiestructuradas y se sometieron a un análisis de contenido. Los datos cuantitativos se extrajeron del libro de indicadores obstétricos y se procesaron mediante un análisis estadístico descriptivo.

**Resultados:**

la correlación de los datos señaló factores determinantes para la actuación de las especialistas: perfil institucional, deficiencias en los flujos de comunicación sobre las directrices del Apice On y limitaciones de los programas de residencia a la hora de articular los preceptos de la educación interprofesional para una práctica colaborativa en obstetricia.

**Consideraciones Finales:**

experimentar procesos de transición organizacional de modelos de atención requiere comunicación horizontal, conciencia, compromiso de los profesionales y definición de roles para la superación de barreras institucionales.

## INTRODUCTION

Historically, especially since the 1970s, Brazilian obstetric care has been marked by the incorporation of technology and the understanding of pregnancy and childbirth from biomedical perspectives. There is still frequent segmentation of the care process, adoption of interventionist practices, and devaluation of women’s autonomy and participation in the childbirth experience, which has rendered them secondary in this scenario^(1-4)^.

In order to reverse this care logic, which has caused dissatisfaction among women regarding the care received, the Ministry of Health (MoH) has been implementing policies in the area of women’s health that value the humanistic principles reported by international organizations. Thus, in 2011, the *Rede Cegonha* was established with the aim of ensuring access, welcoming, and quality in childbirth and birth care^(5)^. Additionally, training and availability of nurse midwives were encouraged, as well as the consolidation of these specialists in the care of low-risk childbirth^(6-8)^.

Shortly after the implementation of *Rede Cegonha*, the Born in Brazil: National Survey on Childbirth and Birth (*Nascer no Brasil: Inquérito Nacional sobre Parto e Nascimento*) was conducted, which described Brazil’s obstetric overview, characterized by a high percentage of cesarean sections and interventionist practices that ignore scientific evidence and reduce the participation of nurse midwives in normal births without dystocia. Based on this portrait of perinatal care, research institutions and universities were recommended to strengthen the involvement and investment of teaching hospitals (TH) in continuing education, aiming at training healthcare professionals for interdisciplinary and teamwork-based practice, in order to increase partnership and collaboration^(9)^.

Thus, given the need and importance of an initiative related to professional training and improvement, the MoH, in partnership with the *Universidade Federal de Minas Gerais*, launched the project for Improvement and Innovation in Care and Teaching in Obstetrics and Neonatology (Apice On - *Aprimoramento e Inovação no Cuidado e Ensino em Obstetrícia e Neonatologia*) in August 2017, with the participation of 97 THs. Among other measures, it aimed to improve clinical training and care management regarding childbirth, birth, and abortion using a care model based on scientific evidence, rights, and principles of humanization. Furthermore, interdisciplinary collaborative work was encouraged by expanding the role of nurse midwives in low-risk childbirth-principles that drive initial movements of change in traditional models of training, care, and management within these institutions^(5,10)^.

Currently, the care provided by nurse midwives in the childbirth and birth process is grounded as a proposal for technocratic model transversalization and is supported by scientific knowledge, which determines their attitude, skill, and provides less interventionist and more qualified care^(2,11,12)^. Therefore, care models that ensure the performance of this specialist present better perinatal outcomes when there is encouragement for the implementation of clearly useful practices and reduction of interventions in low-risk normal births^(2,13,14)^. This type of care may potentially impact maternal and neonatal mortality, preventing 40% of maternal deaths and 26% of neonatal deaths by 2035^(15)^.

Recent studies describe the profile of the 97 THs participating in the Apice On Project and cesarean section rates according to the Robson Classification in these institutions, as well as analyze the challenges of implementing this project in TH in the state of São Paulo, from the perspective of humanization of obstetric care, and report practical experiences during the Apice On Project Advanced Training Course for Nurse Midwives^(16-19)^. However, no research has addressed, to date, the insertion and performance of nurse midwives in the care of low-risk normal births based on one of the expected outcomes of the Apice On Project^(10)^.

Based on the theoretical framework of Afaf Ibrahim Meleis’ Transitions Theory, it is understood that the implementation of the Apice On Project in participating teaching institutions reveals a model of institutional transition in obstetric care. According to this theoretical framework, organizational transition is accompanied by internal changes in institutional structure or dynamics through the adoption of new practices, procedures, technologies, and care models, which ultimately affect the lives of workers in this environment^(20)^. In this regard, the question arises as to how the insertion of nurse midwives in the delivery room of a TH occurred through adherence to the Apice On Project proposal, which previously did not include the presence of these specialists in the childbirth and birth scenario.

## Study relevance

This article is the product of a thesis^(21)^ deposited in the institutional repositor (http://app.uff.br/riuff/handle/1/31507).

## OBJECTIVES

To understand the transition of the inclusion of nurse midwives in the delivery room as part of the Apice On Project in a TH.

## METHODS

### Ethical aspects

The study followed the guidelines of Resolution 466/2012 of the Brazilian National Health Council, with approval from the Ethics Committee. The participating subjects signed the Informed Consent Form, and, to ensure confidentiality and anonymity, they were identified by fictitious codenames represented by the letters C-NM: “care nurse midwife”; UPM-N for “unit production manager nurse”; AM-N for “area manager nurse”; C-OP representing “care obstetric physician”; and AM-OP for “area manager obstetric physician”. These letters were followed by an alphanumeric system according to the order in which they were conducted. The classifications were based on the roles performed by participants during the execution of the Apice On Project.

### Study design

This is a single-case descriptive study supported by the embedded mixed-method design. This approach allows the researcher to include supplementary quantitative data within the traditional qualitative design in order to improve the broader project, represented by the notation system QUAL (+quan)^(22)^.

### Study setting

A maternity hospital of a TH, a reference center for the care of pregnant women with high maternal-fetal risk, located in the municipality of Rio de Janeiro. In March 2018, the institution was selected to join the Apice On Project, concluding its participation in June 2020.

### Methodological procedures

To ensure methodological rigor in the qualitative phase, the COnsolidated criteria for REporting Qualitative research (COREQ) instrument was used as the basis for developing the research report^(23)^.

The data source for the qualitative phase consisted of interviews with professionals who met the following inclusion criteria: being a nurse midwife or daytime on-duty obstetric physician, either a staff member or contractor, who performed care functions in the delivery room from Monday to Friday, considering that the unprecedented incorporation of nurse midwives into the obstetric center staff occurred during weekday daytime shifts; being a nurse midwife or obstetric physician acting as an area and production unit (PU) manager involved in institutional management and implementation of the project. Obstetric physicians and nurse midwives who, during the two years of execution of the policy proposal, were on prolonged medical leave or stopped working in the maternity hospital to take over other institutional roles were not included. Thus, a total of 18 professionals eligible for participation in the study was obtained: one area manager nurse, three PU manager nurses, six on-duty nurse midwives, one area manager obstetric physician, and seven on-duty obstetric physicians. It is important to highlight that there were no refusals among eligible participants and that two professionals-one nurse midwife and one obstetric physician-did not meet the defined criteria and, therefore, did not participate in the study.

The quantitative phase was based on data from the book called “*Indicadores Obstétricos Apice On*”, completed by obstetrics medical residents for each procedure and made available in the Obstetric Center sector of this maternity hospital.

### Data collection and organization

In the qualitative phase, semi-structured interviews were recorded between October 2021 and April 2022 using a smartphone, with prior authorization from participants. Initially, face-to-face contact was established between the researcher and participants on the day they were present at the maternity hospital to explain the purpose of the research. After this initial approach and verbal agreement, individual interviews were scheduled at a convenient date and time, taking place in meeting rooms of the obstetrics department to ensure confidentiality and privacy^(21)^. In this study, intentionally selected participants were included because they represented the totality of eligible subjects. Therefore, the dataset obtained represented sufficient value and validity for understanding the phenomenon, and data closure did not occur based on the theoretical saturation criterion.

The semi-structured script was initially tested and no need for adjustments was identified. The interview included questions regarding professional characterization and guiding questions: What is your understanding of the Apice On Project and its implementation in the maternity hospital? What is your understanding of the insertion and performance of nurse midwives in the delivery room based on the Apice On Project? Do you perceive any change in the routine of obstetric care following the implementation of the project? The interviews were conducted by the lead author, a nurse midwife from the institution and, at the time, a doctoral student in a Graduate Program, with knowledge of qualitative research techniques. During the methodological stages, there was support from the advisor, with expertise in the theoretical and methodological framework.

The quantitative phase began after completion of qualitative data collection and was represented by a cross-sectional study conducted from December 2022 to April 2023. Obstetric data were entered into Epi Info 7^®^ (version 7.2.5.0). However, the period of interest for indicator production corresponded to May 2018 to June 2020, the duration of the Apice On Project implementation in this TH^(21)^.

### Data analysis

The interviews lasted an average of 47 minutes; they were fully transcribed in Microsoft Word^®^ - Microsoft Office and presented to participants for content validation, as recommended by COREQ^(23)^. They were then subjected to content analysis using the thematic modality structured in stages^(24)^. Pre-analysis followed a text skimming process of the constituted *corpus*, resulting from repeated readings by the researcher and impressions conveyed by the messages based on the study assumption. Subsequently, material exploration enabled coding through segmentation, aggregation, and enumeration, determining the units of record. Finally, processing of results and interpretation occurred through grouping record units based on similarity and correspondence among elements. These stages were supported by ATLAS.ti, a qualitative data analysis software^(21)^.

At the end of this analytical process, 1,042 units of meaning, 45 units of record, and 9 thematic cores were obtained, namely: TH maternity hospital profile; multidisciplinary staff professional practice; Apice On Project perception; Apice On Project execution; strategies for project implementation; insertion of nurse midwives in the delivery room; Apice On Project impacts; and professional qualification as improvement of care practice and the collaborative model in obstetrics. After progressive regrouping, they gave rise to three final categories expressing motivations, attitudes, values, beliefs, and tendencies related to the studied topic. This article is based on a subset of the categories: “The teaching hospital maternity” and “Health policy: Improvement and Innovation in Care and Teaching in Obstetrics and Neonatology (Apice On) in a teaching hospital”, extracted from the thesis entitled “*A transição da inserção de enfermeiras obstétricas em sala departo de HE como estratégia do projeto Apice On para a ressignificação do parto e nascimento: um estudo de caso*”, presented to the Academic Program in Health Care Sciences at *Universidade Federal Fluminense* ^(21)^.

In the quantitative phase, the sample consisted of 2,113 cases of patients receiving obstetric care with gestational age ≥ 22 weeks who underwent vaginal delivery or cesarean section. Subsequently, this database was exported and subjected to descriptive statistical analysis with calculation of absolute and relative frequencies using Statistical Package for the Social Sciences (SPSS^®^).

The study variables presented in this section were individual and obstetric characteristics (age group, parity, previous cesarean section, high-risk or low-risk pregnancy, type of delivery). For the sample of vaginal births, the Robson Classification group, the use of non-pharmacological methods for pain relief, and the professional assisting the birth were used as variables. The definition of these variables was based on the indicator matrix proposed by the Apice On Project in its component of obstetric care qualification^(21)^.

At baseline, analysis was conducted for the five months prior to adherence to the Apice On Project, from December 1, 2017, to April 30, 2018, when recording of information related to the use of non-pharmacological pain relief methods during labor began in the vaginal birth registry book. Thus, the sample included 238 women with gestational age ≥ 22 weeks who underwent vaginal delivery^(21)^.

## RESULTS

The excerpt of the thesis presented in this article describes the content expressed by participants regarding the implementation of the Apice On policy proposal in this TH maternity hospital. It refers to a period of organizational transition, represented by the insertion of nurse midwives in the delivery room with the purpose of providing care for low-risk births, and the consequent challenges along this process.

As an initial analysis of interviews from the qualitative phase of the study, it is relevant to present the participants’ profile. The group of professionals working in the maternity hospital shows that 12 have *lato sensu* training in obstetrics residency, 11 completed *stricto sensu* graduate education at the master’s level, and five at the doctoral level. Regarding length of service in this institution, two had up to 5 years, four between 6 and 10 years, one between 11 and 15 years, eight between 16 and 20 years, one between 21 and 25 years, and two with more than 26 years^(21)^.

Concerning the characterization of the sample of women who underwent obstetric procedures, most were aged between 20 and 34 years, multiparous, and without previous cesarean section. Slightly more than half were composed of women without gestational risk; however, among those classified within this group, a large portion presented only one complication, with fetal malformation being the most prevalent, followed by hypertension (Table 1)^(21)^.

**Table 1:** Individual and obstetric characteristics of women with gestational age ≥ 22 weeks undergoing obstetric procedures in a teaching hospital, Rio de Janeiro, Rio de Janeiro, Brazil, May 2018 to June 2020

Variables	n	%
**Age group (n = 2,039)**		
≤ 15 years	41	2.0
16 to 19 years	251	12.3
20 to 34 years	1,329	65.2
≥ 35 years	418	20.5
**Paritye (n = 2,095)**		
Nulliparous	889	42.4
Multiparous	1,206	57.6
**Previous cesarean section (n = 2,087)**		
Yes	538	25.8
No	1,549	74.2
**High-risk pregnancy (n = 1,872)**		
Yes	794	42.4
No	1,078	57.6
**Number of complications (n = 959)**		
1 complication	794	82.8
2 complications	137	14.3
3 or more complications	28	2.9
**Types of complications (n = 830)**		
IUGR^*^	35	4.2
Gestational diabetes	59	7.1
Pre-gestational diabetes	1	0.1
Severe chronic disease	4	0.4
PA^**^	1	0.1
Eclampsia	2	0.2
Hypertension	202	24.3
Infections	113	14.1
FM^***^	377	45.2
Placenta previa	0	0
Other	36	4.3

* IUGR - Intrauterine Growth Restriction;

** PA - Placental Abruption;

*** FM - Fetal Malformation.

**Chart 1:** Data integration and generation of inferences between qualitative and quantitative data

The maternity ward of a teaching hospital and its obstetric care according to Apice On	Qualitative research	Quantitative research	Inferences, convergences, and divergences
Apice On project execution Results not achieved according to the care qualification component • Low-risk vaginal births assisted by nurse midwives	I think we could have more of this sharing with the staff, not only when I need it, I will only share with you because I have an emergency there. […] it is not the scenario we imagined, the performance of a nurse midwife one hundred percent […] performing low-risk births. […] despite the peculiarities of the service, we have low-risk pregnant women who could perfectly be followed by obstetric nursing [...]. (C-NM/1) [...] there was no planning on how this division with the medical staff would be. I think if this had been better structured, gradually people would get used to the idea of obstetric nursing being active. (C-NM/4) If we had started proposing a shared space, I think it would have been much better accepted than births exclusively assisted by nurses as proposed in the project. Given our low number and the profile of our women, we could perform the births. (C-NM/5) I find it very complicated when things are only on paper and suddenly appear! And you think, “What? Who is it? How is it? How will it work? Are there limits? Are there no limits?” […] no one knew how obstetric nursing was being introduced. Did it have to be this difficult? No! And those who arrive, poor them! They also come in defensive mode because they are not being well received! (C-OP/9) I think this difficulty was due to this lack of dissemination, lack of discussion […] suddenly two nurse midwives arrived and were placed there, you know?! And then everyone was like, “Now what?” (C-OP10) But if I also agreed with that situation, what would happen to my residents? One birth per day! Who would it be for? […] being a maternity hospital that performs 100 births/month and, of these, 50% are cesarean sections, we have three medical residency classes […] and then I incorporate corporatism, understanding that, within this maternity hospital, this obstetric care model by nurse midwives would take space from my resident. [...]. (C-OP/12) And then physicians would arrive at the obstetric center during labor and delivery care. As most of them have no experience with obstetric nursing, they were afraid of the nurse acting. I don’t know if they think we are not competent or if they do not understand our scope. Because some even say, “Oh! But you will provide care, but if it gets complicated, you call us, so it doesn’t make sense!” […] they have this view that nurses must always be alongside the physician, performing something that is somehow “authorized” by the physician. (AM-N/14) [...] residents are already more used to working with nurse midwives outside. Younger staff as well. But older staff did not even know what the competencies of nurse midwives are. […] so, it was often seen as if we did not have the necessary qualification. Like a stopgap, you know?! (UPM-N/18)	- 51.9% of births occurred via cesarean section and 48.1% via vaginal delivery. - Of the 43.9% of normal deliveries with usual risk, 33.2% corresponded to group 1 and 31.7% to group 3 of the Robson Classification. These represent the potential for normal deliveries for the physiological evolution of labor. - 75.7% of women used some type of non-pharmacological method for pain relief. In a baseline of the months prior to joining the project, 58.3% of postpartum women benefited from this good obstetric practice. - 1.1% of normal deliveries were assisted by nurse midwives in the delivery room of this teaching hospital.	Quantitative data plays a supporting role over qualitative data. The inclusion of nurse midwives in the delivery room and the assistance in normal deliveries of habitual risk expanded the availability of non-pharmacological methods for pain relief. However, their full involvement in habitual risk deliveries, as per the project guidelines, presented a set of factors that led to deviations in achieving the project’s proposed outcome. - The institutional profile characteristic of a referral unit for maternal and fetal risk pregnancies determined the reduced number of pregnant women classified as habitual risk. - Furthermore, the lack of communication between managers and the care staff regarding the project guidelines affected the planning of obstetric care during childbirth and the responsibilities and competencies of each professional from the perspective of the shared model. - The important question regarding the harmonization between the mission of a teaching hospital and the training process of medical residents.

The combination of data through incorporation is presented in Chart 0031 by the joint display, a framework that integrates and groups them visually. The display contains the first column related to the implementation points of the care qualification component described in the Apice On Project; the second column consists of the organization of the most representative participant narratives (qualitative domain); the third column presents some indicators (quantitative domain); and, finally, the fourth specifies the inferences or insights generated as a result of complementarity, divergences, and convergences of the findings^(21)^.

## DISCUSSION

In relation to the insertion of nurse midwives, it is inferred that there was no participation of these professionals in the care of low-risk vaginal births in the delivery room of this TH, which represents a result not achieved according to the obstetric care qualification component, which established a target of 70% for this indicator, according to the obstetric care indicator matrix of the Apice On Project. It is worth noting that this finding contrasts with an evaluative study that identified an increase in the percentage of births assisted by nurse midwives from 13.7% in the Born in Brazil (*Nascer no Brasil*) study to 24.6% in the assessment of *Rede Cegonha* in southeastern Brazil^(25)^.

As evidenced, the presence of nurse midwives in the delivery room contributed to an increase in the use of non-pharmacological methods for pain relief when compared to data prior to the implementation of the project. In this sense, even though it is a maternity hospital with a high maternal-fetal risk profile, the adoption of a multidisciplinary care strategy with the presence of these specialists is valued, as they are capable of favoring the physiological progression of labor, the implementation of good obstetric practices, the reduction of interventions during labor, and cesarean section rates. Moreover, they establish relational care through attitudes of availability, empathy, welcoming, and sensitivity^(26)^, which improve maternal satisfaction regarding the experience of vaginal birth^(6,13-15)^.

However, despite this evidence, the study identified barriers to the performance of these professionals, structured around three central and interrelated axes. The first is based on the care profile of a maternity hospital with maternal and fetal complexity, resulting in a reduced number of women classified as low risk, who meet the legal criteria for follow-up by nurse midwives during labor^(6)^. This characteristic, to some extent, justified the lack of performance of these specialists in the delivery room.

In the field of women’s health, THs are considered the main scientific structures for knowledge transmission and are reference centers for high-risk pregnancies, which reinforces the biomedical assumption that the female body requires control and intervention to achieve favorable biological outcomes^(3,4,27)^. These particularities influence the high rates of invasive procedures, cesarean sections, and the low incorporation of nurse midwives in these institutions participating in the Apice On Project^(16,17)^.

In addition to this institutional profile, the fragility of communication between managers and the care staff regarding the project guidelines and the proposal of a collaborative model of obstetric care was identified as a second key factor in this organizational transition process. From the perspective of some participants, the top-down flow of organizational communication and policy directives led to distortions along this communication chain due to individual interpretations influenced by cultural differences, experiences, interests, and expectations^(28)^.

These aspects may have induced processes of selection, rejection, acceptance, and interpretation of the message, significantly affecting participants’ awareness of the implementation of the Apice On Project. This is because awareness is considered an important property of the transition experience, acquired through knowledge and recognition of the process, reflected in the degree of congruence between what is known and the responses involved. In short, to be in transition, one must have some awareness of the ongoing changes^(20)^.

Consequently, the level of engagement in this organizational transition-understood as the degree of interest demonstrated by an individual in being involved in the process-was found to be deficient. Therefore, it can be inferred that the level of awareness, achieved through adequate communication, influenced the level of professional engagement in the development of this ministerial initiative^(20)^. In this perspective, a gap between the political and practical fields was identified, representing a limitation in articulating commitments and attitudes by managers to translate the theoretical elements of the Apice On Project into internal institutional actions toward a new obstetric care model in this TH^(21)^.

Thus, the importance is highlighted not only of continuous qualification of managers to face problems and challenges throughout the health management process, but also of promoting strategies for shared management and spaces for dialogue^(10)^. Similarly, studies have reported management difficulties in engaging and integrating staff in the development of the Apice On Project, as well as barriers to disseminating the information necessary for understanding its meanings, goals, and competencies to be mobilized by the care staff^(18)^.

It is also important to emphasize the communication deficit regarding this ministerial proposal, mainly pointed out by the obstetric staff, as an amplifying factor of resistance to the insertion of nurse midwives in the delivery room. In this transition process of care models, it is common for physicians to be concerned about the experience and capability of nurse midwives and to have doubts regarding the definition of responsibilities, as they consider childbirth management to be exclusively within their technical domain. On the other hand, nurse midwives consider themselves responsible for women’s care and disagree that their actions should be restricted to bureaucratic roles or subordinated positions^(7,29-31)^.

In this environment characterized by hierarchy, divergent opinions, and opposition between care models-technocratic and humanistic-nurse midwives face a traditionally physician-centered culture and struggle for autonomy and recognition of their competencies. In this sense, professional conduct demonstrates a lack of alignment, particularly among the medical staff, in implementing collaborative practices and evidence-based obstetric care^(1,8,27,32,33)^.

Additionally, according to transition theory, this work context-permeated by personal, cultural, and socioeconomic meanings of each individual or professional group-may hinder a healthy organizational transition. Furthermore, the level of prior preparation, knowledge, and personal expectations may interfere with understanding the new roles of obstetric physicians and nurse midwives within this proposed model of care in this TH^(20)^.

Consequently, the last determining axis identified in this study for the lack of performance of nurse midwives was the Medical Residency Program in Gynecology and Obstetrics, which carries significant institutional weight. The reduced number of low-risk vaginal births, combined with the possibility of sharing these with nurse midwives, intensified discussions and conflicts. This represents a conflictive dynamic that hinders the formulation of proposals from both sides to advance the ministerial strategy^(21)^.

These relationships are contextualized within traditional teaching models, in which the female body becomes an object of training through repetitive and often inadequate use of techniques and procedures not aligned with evidence. Ultimately, a relationship is established in which the quality of learning is directly linked to the number of procedures performed by students^(4,34)^.

Thus, this care dynamic, which values uniprofessional practice, runs counter to interprofessional education (IPE) in health, a movement aimed at improving individual and collective skills, strengthening communication, and teamwork. These measures reduce care fragmentation, increase mutual respect among professionals, and consequently reduce negative attitudes among students toward other professional categories^(35,36)^.

Therefore, IPE is recognized as an important strategy to be adopted in TH participating in the Apice On Project to achieve expected outcomes in the field of teaching qualification: the formalization of educational strategies that enhance collaborative learning among students and the articulation between care, education, and integrated work among multidisciplinary staff^(10,18)^. These guidelines strengthen the training of new professionals “forged in the expertise of good obstetric practices”, who become key elements in reconstructing the childbirth care model^(34)^.

It is observed, therefore, that the challenges faced by nurse midwives in the daily routine of obstetric centers-where preference for childbirth management lies with obstetricians-are present both in care, due to deficient integration among obstetric physicians, nurse midwives, and medical and nursing residents, and in training, due to disputes and segregation during practice and learning in real scenarios. It is believed that the coexistence of medical and nursing residents in the delivery room provides a valuable interprofessional exchange of knowledge, capable of enhancing training processes. Additionally, nurse midwives may demonstrate humanized care practices, while residents may incorporate new perspectives throughout their training process, potentially reducing resistance to paradigm changes in childbirth and birth care^(21)^.

It is necessary to emphasize that advancing toward a collaborative model in obstetrics-one that includes the participation of nurse midwives in the care of low-risk labor and childbirth, with activation, support, and management of complications by obstetric physicians and in high-risk pregnancies^(14)^-requires close collaboration between these specialized professionals. Therefore, attitudes of dialogue, trust, and respect are expected in the coordinated execution of actions, so that healthcare work processes are developed with shared objectives and goals, under an expanded conception of the health-disease process. Models that ensure organizational changes toward quality healthcare and democratization of work environments are essential^(35,37,38)^.

It is considered that, five years after the conclusion of the Apice On Project in this TH, a period of balance in relationships has been achieved, with a reduction in conflicts between obstetricians and nurse midwives. From the perspective of Meleis’ theory, time is a property of the transition process, with a beginning marked by an identifiable starting point-in this case, adherence to the ministerial proposal-which extended from the first signs of anticipation, perception, or demonstration of change. This was followed by a period of instability, confusion, and distress, particularly regarding feelings of frustration and powerlessness experienced by nurse midwives as a result of the reconstruction of their roles in this scenario. The healthy completion of this organizational transition process was marked by a new beginning or period of stability, resulting from the conclusion of the political project. At this point, professionals achieved mastery of roles and behavioral patterns necessary to manage situations within this new relational configuration, consolidating the presence of nurse midwives in the delivery room^(20,21)^.

Thus, throughout this process of recognizing the multiple adversities in achieving the purpose of the Apice On Project regarding the care of low-risk vaginal births by nurse midwives, individual and collective reflections were fostered on the ideal collaborative model to be implemented in this TH. The incorporation of a collaborative model of obstetric care, although recognized in many institutions as feasible and satisfactory, may not be the best approach for others. Therefore, based on the experience of this TH, it is recommended to move toward the construction of care models adapted to specific contexts and obstetric profiles, always considering the interprofessional perspective of obstetric care^(21)^.

### Study limitations

The impossibility of encompassing the perception of other actors within the multidisciplinary staff who may influence the performance of nurse midwives is highlighted. The manual recording of information in the indicator logbook also represents a limitation, given the possibility of data entry errors. Additionally, the infeasibility of faithfully reproducing these results for the other 97 THs participating in the Apice On Project is pointed out, since, as mentioned, there are particularities related to the institutional context investigated.

### Contributions to nursing, health, or public policies

The knowledge, description, and analysis of the insertion of nurse midwives within a context of institutional transition of the obstetric care model in TH make it possible to identify factors that affect the practice and autonomy of this professional during care praxis in low-risk vaginal births. Thus, important strategies and articulations are suggested at relational, managerial, and institutional levels, capable of enabling and expanding the incorporation of these specialists-whose training is grounded in scientific evidence and humanistic principles-into multidisciplinary staff. It is expected, therefore, to improve perinatal care and, especially, to make these settings responsible for shaping collaborative attitudes and practices among future professionals.

## FINAL CONSIDERATIONS

This study described a process of organizational transition fostered by the participation of a TH in the Apice On Project and the consequent inclusion of nurse midwives in the delivery room. It represents an unprecedented initiative, given the previous absence of these specialists on a permanent basis in this setting.

However, it is important to highlight that key elements affected the lack of formalization of an institutional protocol that would validate the legal attributions of these professionals in the follow-up of low-risk vaginal births. These include: the maternal-child care profile; deficient communication between managers and on-duty staff, considered an important managerial resource for engagement and involvement of all staff in institutional policy; lack of knowledge among obstetricians regarding the competencies and responsibilities of each professional within a shared model of obstetric care; and the institutional mission of training future professionals through residency programs.

The conditions that interfered with the quality of this transition experience for participants-such as meanings, expectations, and level of knowledge-led to a new care configuration between nurse midwives and obstetricians in the delivery room, which remains balanced to this day. The staff began to understand the characteristics of this coexistence and the scope of roles, as nurse midwives took over a secondary role in this relational dynamic with obstetric physicians regarding the management of uncomplicated vaginal births, which resulted in the insufficiency of their role.

Thus, by understanding the process of inserting nurse midwives in the delivery room based on the Apice On Project in a TH, the close interdependence between organizational transition processes and the dimensions related to communication, interpersonal relationships, and professional conceptions of childbirth and birth is revealed. It also highlights the time required to define new roles to be incorporated by obstetricians and nurse midwives toward the implementation of new obstetric care models.

In this transitional context, it is emphasized that the value of the presence and performance of this professional goes beyond the recognition of the physiology of childbirth to ensure the implementation of recommended practices, as it also includes providing specialized technical and relational care. Therefore, discussion and expansion of care models that ensure the insertion of nurse midwives in multidisciplinary contexts are encouraged as a permanent agenda in women’s health policies, with temporal and territorial scope. This is a fundamental measure, as these are healthcare professionals considered essential in providing safe and qualified care, especially in highly specialized and high-complexity settings.

## Data Availability

The research data are available only upon request.
