# Supplementary material for: Transition of the insertion of nurse midwives in the delivery room of a teaching hospital
Source: Rev Bras Enferm. 2026 Oct 19;79:e20250190. doi: 10.1590/0034-7167-2025-0190 (PMC13679595; doi:10.1590/0034-7167-2025-0190)
Supplement: Translation

# Translation: A transição da inserção de enfermeiras obstétricas em sala de parto de hospital de ensino

**DOI:** 10.1590/0034-7167-2025-0190pt

**Authors:** Thalita Rocha Oliveira, Valdecyr Herdy Alves

**Affiliations:** I Instituto Nacional de Saúde da Mulher, da Criança e do Adolescente Fernandes Figueira/Fiocruz. Rio de Janeiro, Rio de Janeiro. Brasil; II Universidade Federal Fluminense. Niterói, Rio de Janeiro, Brasil

**Keywords:** Enfermeiras Obstétricas, Hospitais de Ensino, Sala de Parto, Serviços de Saúde Materno Infantil, Política de Saúde.

## Abstract

**Objetivos:**

compreender a transição da inserção da enfermeira obstétrica em sala de parto a partir do Projeto Apice On em hospital de ensino.

**Métodos:**

estudo de método misto - Projeto Incorporado Concomitante. Os dados qualitativos foram coletados com 18 profissionais de uma maternidade do município do Rio de Janeiro, por meio de entrevistas semiestruturadas, e submetidos à análise de conteúdo. Os dados quantitativos foram extraídos do livro de indicadores obstétricos e processados por análise estatística descritiva.

**Resultados:**

a correlação dos dados apontou fatores determinantes para a atuação das especialistas: perfil institucional, deficiência dos fluxos comunicacionais sobre as diretrizes do Apice On e limitação dos programas de residência em articular preceitos da educação interprofissional para uma prática colaborativa em obstetrícia.

**Considerações Finais:**

vivenciar processos de transição organizacional de modelos de cuidado requer comunicação horizontalizada, consciência, engajamento dos profissionais e definição de papéis, para a transposição de barreiras institucionais.

## INTRODUÇÃO

Historicamente, sobretudo a partir da década de 70, a assistência obstétrica brasileira tem sido marcada por incorporação de tecnologia e compreensão da gestação e do parto sob perspectivas biomédicas. Há ainda frequente segmentação do processo de cuidado, adoção de práticas intervencionistas, desvalorização da autonomia e participação feminina na vivência da parturição, o que a tornou coadjuvante neste cenário^(1-4)^.

Com intuito de reverter esta lógica assistencialista, causadora de insatisfação das mulheres quanto ao cuidado recebido, o Ministério da Saúde (MS) vem implementando políticas na área da saúde da mulher que valorizam os preceitos humanísticos relatados por organismos internacionais. Assim, em 2011, institui-se a Rede Cegonha, com o objetivo de garantir acesso, acolhimento e qualidade na atenção ao parto e nascimento^(5)^. Adicionalmente, fomentou-se a capacitação e oferta de enfermeiras obstétricas, bem como a consolidação destas especialistas na assistência ao parto de risco habitual^(6-8)^.

Logo após a implantação da Rede Cegonha, foi realizado o Inquérito Nacional sobre parto e nascimento - Nascer no Brasil, o qual descrevia o panorama obstétrico do país, pautado em elevado percentual de cirurgias cesarianas e práticas intervencionistas, que ignoram as evidências científicas e reduzem a participação de enfermeiras obstétricas na realização de partos normais sem distocia. Com base neste retrato da assistência perinatal, foi recomendado às Instituições de Pesquisa e Universidades reforçar o envolvimento e investimento dos Hospitais de Ensino (HE) na educação permanente, tendo em vista a formação de profissionais de saúde para o trabalho interdisciplinar e em equipe, no sentido de aumentar a parceria e a colaboração^(9)^.

Assim, diante da necessidade e importância de uma iniciativa relacionada à formação e ao aprimoramento profissional, o MS, em parceria com a Universidade Federal de Minas Gerais (UFMG), lançou o Projeto de Aprimoramento e Inovação no Cuidado e Ensino em Obstetrícia e Neonatologia (Apice On), em agosto de 2017, com a participação de 97 HE. Propunha-se, dentre outras medidas, melhorar a formação clínica e a gestão do cuidado em relação ao parto, nascimento e abortamento utilizando um modelo de cuidado baseado em evidências científicas, nos direitos e princípios da humanização. Ademais, incentivava-se o trabalho colaborativo interdisciplinar a partir da ampliação da atuação de enfermeiras obstétricas no parto de risco habitual, princípios estes que impulsionam movimentos iniciais de mudanças nos modelos tradicionais de formação, atenção e gestão junto a essas instituições^(5,10)^.

Atualmente, o cuidado da enfermeira obstétrica no processo do parto e nascimento fundamenta-se como uma proposta de transversalização ao modelo tecnocrata e sustenta-se no conhecimento científico, o qual determina a sua atitude, habilidade e proporciona um cuidado menos intervencionista e mais qualificado^(2,11,12)^. Portanto, modelos de cuidado que garantem a atuação desta especialista apresentam melhores resultados perinatais quando há incentivo à implantação de práticas notadamente úteis e à redução das intervenções nos partos normais de risco habitual^(2,13,14)^. Este tipo de assistência pode impactar potencialmente na mortalidade materna e neonatal, evitando 40% das mortes maternas e 26% de mortes neonatais até 2035^(15)^.

Estudos recentes descrevem o perfil dos 97 HE participantes do Projeto Apice On e taxas de cesáreas segundo a Classificação de Robson realizadas nestas instituições, bem como analisam os desafios da implantação deste projeto em HE do estado de São Paulo, sob a perspectiva da humanização da assistência obstétrica, e relatam as experiências práticas vivenciadas durante a realização do Curso de Aprimoramento para Enfermeiras Obstétricas (CAEO) do Projeto Apice On^(16-19)^. Contudo, nenhuma pesquisa abordou, até este momento, a inserção e atuação de enfermeiras obstétricas na assistência a partos normais de risco habitual a partir de um dos resultados esperados pelo Projeto Apice On^(10)^.

Sob o referencial da teoria das transições de Afaf Ibrahim Meleis, compreende-se que a implantação do projeto Apice On nas instituições de ensino participantes revela um modelo de transição institucional da assistência obstétrica. Com base nesse referencial teórico, a transição organizacional vem acompanhada de mudanças internas na estrutura ou dinâmica institucional, mediante a adoção de novas práticas, procedimentos, tecnologias e modelos de cuidado, que acabam por afetar a vida dos trabalhadores neste ambiente^(20)^. Nesse sentido, questiona-se como ocorreu a inserção de enfermeiras obstétricas em sala de parto de um HE por meio da adesão à proposta do Projeto Apice On, que até então não contava com a presença destas especialistas no cenário de parto e nascimento.

## Relevância do estudo

Este artigo é produto da Tese^(21)^ depositada no repositório institucional (http://app.uff.br/riuff/handle/1/31507).

## OBJETIVOS

Compreender a transição da inserção da enfermeira obstétrica em sala de parto a partir do Projeto Apice On em Hospital de Ensino.

## MÉTODOS

### Aspectos éticos

O estudo seguiu as diretrizes da Resolução nº466/2012 do Conselho Nacional de Saúde, com aprovação do Comitê de Ética. Os sujeitos participantes assinaram o Termo de Consentimento Livre e Esclarecido e, para garantir o sigilo e anonimato, foram identificados por codinomes fictícios representados pelas letras EO-A: “Enfermeira obstétrica assistencial”; E-GUP para “Enfermeira Gestora de Unidades de Produção”, E-GA para “Enfermeira Gestora da Área de Atenção”; MO-A em representação ao “Médico obstetra assistencial” e MO-GA ao “Médico obstetra Gestor da Área de Atenção”. Tais letras foram seguidas de um sistema alfanumérico, conforme a ordem de execução das mesmas. As classificações basearam-se nas funções desempenhadas pelos participantes durante a execução do projeto Apice On.

### Tipo de estudo

Estudo de caso único, do tipo descritivo, sustentado pelo Projeto Incorporado do Método Misto. Tal abordagem possibilita ao pesquisador incluir dados suplementares quantitativos ao projeto tradicional qualitativo, no sentido de proporcionar melhorias ao projeto maior e representado pelo sistema de notação QUAL(+quan)^(22)^.

### Cenário do estudo

Uma maternidade de HE, referência para atendimentos de gestantes com alto risco materno-fetal, localizada no município do Rio de Janeiro. Em março de 2018, a instituição foi selecionada para aderir ao Projeto Apice On, finalizando sua participação em junho de 2020.

### Procedimentos metodológicos

Para contemplar o rigor metodológico da etapa qualitativa, utilizou-se o instrumento *Consolidated Criteria for Reporting Qualitative Research* (COREQ) como base para o desenvolvimento do relatório de pesquisa^(23)^.

A fonte de dados da etapa qualitativa consistiu em entrevistas a profissionais que atenderam aos seguintes critérios de inclusão: ser enfermeira obstétrica ou médico obstetra plantonista diurno, servidor ou contratado, que desempenhou funções de assistência em sala de parto de segunda a sextafeira, visto que a inédita incorporação de enfermeiras obstétricas na equipe do Centro Obstétrico ocorreu nos plantões diurnos durante a semana; ser enfermeira obstétrica ou médico obstetra gestor de Área e Unidade de Produção (UP) envolvido com a gestão institucional e implantação do projeto. Não foram incluídos médicos obstetras e enfermeiras obstétricas que, durante os dois anos de execução da proposição política, foram afastados por licença-médica prolongada ou deixaram de atuar na maternidade para assumir outras funções institucionais. Sendo assim, obteve-se um total de 18 profissionais elegíveis para participação do estudo: 01 (uma) enfermeira gestora de área, 03 (três) enfermeiras gestoras de UP, 06 (seis) enfermeiras obstétricas plantonistas, 01 (um) médico obstetra gestor de área e 07 (sete) médicos obstetras plantonistas. Importante salientar a ausência de recusa de participação dos selecionáveis e a presença de 02 (dois) profissionais, sendo 01(uma) enfermeira obstétrica e 01(um) médico obstetra, que não atenderam aos critérios delimitados e, portanto, não participaram do estudo.

A etapa quantitativa apoiou-se nos dados provenientes do livro denominado Indicadores Obstétricos Apice On, preenchido pelos residentes médicos de obstetrícia a cada procedimento e disponibilizado no setor Centro Obstétrico desta maternidade.

### Coleta e organização dos dados

Na etapa qualitativa, as entrevistas semiestruturadas foram gravadas no período de outubro de 2021 a abril de 2022, em aparelho *smartphone*, mediante autorização prévia dos participantes. Inicialmente, realizou-se contato de forma presencial entre a pesquisadora e os participantes no dia em que estes se encontravam na maternidade, a fim de expor o propósito da pesquisa. Após esta aproximação inicial e verbalização do aceite, foi agendada, em dia e horário oportuno, a entrevista individual, que aconteceu em salas de reuniões do departamento de obstetrícia, por garantirem sigilo e privacidade^(21)^. Nesta pesquisa, os participantes delimitados de maneira intencional foram incluídos no estudo, pois representavam a totalidade dos sujeitos aptos. Portanto, o conjunto de dados obtidos representou o valor e a validade para a compreensão do fenômeno o que, por conseguinte, determinou que o encerramento dos dados não ocorresse por meio do critério de saturação teórica.

O roteiro semiestruturado foi inicialmente testado e identificou-se ausência de necessidade de adequação do mesmo. A entrevista contava com algumas perguntas a respeito da caracterização profissional e questões norteadoras: “Qual é a sua compreensão sobre o projeto Apice On e sua execução na maternidade?”; “Qual o seu entendimento sobre a inserção e atuação das enfermeiras obstétricas na sala de parto a partir do Projeto Apice On?” e “Você compreende se houve alguma mudança na rotina de cuidado obstétrico a partir da execução do projeto?”. As entrevistas foram realizadas pela autora principal, enfermeira obstétrica desta instituição e, na época, aluna de Programa de Pós-Graduação (nível Doutorado) com conhecimento em técnicas de pesquisa qualitativa. Durante as etapas metodológicas, houve apoio do orientador, com expertise no referencial teórico e metodológico.

A etapa quantitativa iniciou-se após findada a coleta de dados do método qualitativo, representada por um estudo do tipo transversal, que ocorreu de dezembro de 2022 a abril de 2023. Os dados obstétricos foram lançados no *software Epi Info 7^®^* (versão 7.2.5.0). Contudo, o período de interesse para a produção dos indicadores correspondeu aos meses de maio de 2018 a junho de 2020, tempo de execução do Projeto Apice On nesta maternidade de HE^(21)^.

### Análise dos dados

As entrevistas tiveram tempo médio de duração de 47 minutos; foram transcritas na íntegra em programa de texto *Microsoft Word^®^* - *Microsoft Office* e apresentadas aos depoentes para validação do conteúdo, conforme indicação do COREQ^(23)^. Em seguida, passaram por análise de conteúdo na modalidade temática estruturada em etapas^(24)^. A pré-análise seguiu o movimento de leitura flutuante do *corpus constituído*, consequente das ações de leituras e releituras da pesquisadora e impressões transmitidas pelas mensagens em função do pressuposto do estudo. Na sequência, houve exploração do material, que possibilitou a codificação por recorte, agregação e enumeração e determinou as unidades de registro. Por fim, o tratamento dos resultados obtidos e interpretação ocorreu mediante o agrupamento de unidades de registro em razão da semelhança e correspondência entre estes elementos. Essas etapas foram viabilizadas via *software* de análise de dados qualitativos *ATLAS.ti* ^(21)^.

Dessa maneira, ao final deste processo analítico foram obtidas 1042 unidades de significação, 45 unidades de registro e 09 núcleos temáticos, quais sejam: o perfil da maternidade de HE; a prática profissional da equipe multidisciplinar; a percepção do projeto Apice On; a execução do projeto Apice On; as estratégias para execução do projeto Apice On; a inserção da enfermeira obstétrica em sala de parto; impactos do projeto Apice On; e a qualificação profissional como aprimoramento da prática de cuidado e o modelo colaborativo em obstetrícia. Após reagrupamento progressivo, deram origem a três categorias terminais, que expressam as motivações das opiniões, atitudes, valores, crenças e tendências daquele tema estudado. O presente artigo baseia-se em um recorte das categorias: “A maternidade de Hospital de Ensino” e “A política de saúde: Aprimoramento e inovação no cuidado e ensino em obstetrícia e neonatologia (Apice On) em HE” extraídas da tese intitulada “A transição da inserção de enfermeiras obstétricas em sala de parto de HE como estratégia do projeto Apice On para a ressignificação do parto e nascimento: um estudo de caso”, apresentada ao Programa Acadêmico em Ciências do Cuidado em Saúde da Universidade Federal Fluminense^(21)^.

Na etapa quantitativa, a amostra constituiu-se de 2.113 casos de pacientes em atendimentos obstétricos com idade gestacional ≥ 22 semanas submetidas a parto normal e cirurgia cesariana. Posteriormente, este banco de dados foi exportado e submetido à análise estatística descritiva com cálculo de frequências absolutas e relativas pelo pacote estatístico *Statistical Package for the Social Science* (SPSS)^®^.

As variáveis do estudo apresentadas neste recorte foram as características individuais e obstétricas (faixa etária, paridade, parto cesariana prévio, gestação de alto risco ou risco habitual, tipo de parto). Para a amostra de nascimento por parto normal, utilizaram-se, como variáveis, a Classificação do grupo de Robson, o uso de métodos não farmacológicos para alívio da dor e o profissional que assistiu ao parto. A delimitação destas variáveis foi pautada na matriz de indicadores proposta pelo projeto Apice On em seu componente, qualificação da atenção obstétrica^(21)^.

Na linha de base, realizou-se análise dos cinco meses anteriores à adesão ao projeto Apice On, no período de 1 dezembro de 2017 a 30 de abril de 2018, momento em que se iniciou o registro de informações relacionadas ao uso de métodos não farmacológicos para alívio da dor durante o trabalho de parto no livro de registros de partos normais. Sendo assim, compuseram a amostra 238 mulheres com idade gestacional ≥ 22 semanas submetidas a parto normal^(21)^.

## RESULTADOS

O recorte da tese apresentado neste artigo descreve o conteúdo expresso pelos participantes quanto à execução da proposição política do Apice On nesta maternidade de HE. Trata-se de um período de transição organizacional, representado pela inserção da enfermeira obstétrica em sala de parto com o propósito de prestar assistência a partos de risco habitual, e consequentes desafios nesse caminhar.

Como análise inicial das entrevistas da etapa qualitativa da pesquisa, convém apresentar o perfil dos participantes. O corpo de profissionais que atuam na maternidade mostra que 12 (doze) apresentam formação *lato sensu* na modalidade residência em obstetrícia, 11 (onze) concluíram a pós-graduação *stricto sensu* curso mestrado e 5 (cinco) curso doutorado. Quanto ao tempo de atuação nesta instituição, 2 (dois) possuíam até 5 anos, 4 (quatro) entre 6 e10 anos, 1 (um) entre 11 e15 anos, 8 (oito) entre 16 e20 anos, 1 (um) entre 21 e 25 anos e 2 (dois) com mais de 26 anos^(21)^.

Sobre a caracterização da amostra de mulheres que realizaram procedimentos obstétricos, a maioria se encontrava na faixa etária dos 20 a 34 anos, multíparas e sem parto cesárea prévio. Pouco mais da metade era composta por gestantes sem risco gestacional, mas, daquelas que se enquadravam neste critério, grande parcela apresentou apenas uma complicação, sendo a malformação fetal a mais prevalente, seguida da hipertensão arterial (Tabela 1)^(21)^.

**Tabela 1:** Características individuais e obstétricas de mulheres com idade gestacional ≥ 22 semanas submetidas a procedimentos obstétricos em um Hospital de Ensino, Rio de Janeiro, Rio de Janeiro, Brasil, maio 2018 a junho 2020

Variáveis	n	%
**Faixa etária (n = 2.039)**		
≤ 15 anos	41	2,0
16 a 19 anos	251	12,3
20 a 34 anos	1329	65,2
≥ 35 anos	418	20,5
**Paridade (n = 2.095)**		
Nulípara	889	42,4
Multípara	1.206	57,6
**Parto cesáreo prévio (n = 2.087)**		
Sim	538	25,8
Não	1549	74,2
**Gestação de alto risco (n = 1.872)**		
Sim	794	42,4
Não	1078	57,6
**Quantidade de complicações (n = 959)**		
1 complicação	794	82,8
2 complicações	137	14,3
3 ou mais complicações	28	2,9
**Tipos de complicações (n = 830)**		
CIUR^*^	35	4,2
Diabetes gestacional	59	7,1
Diabetes pré-gestacional	1	0,1
Doença crônica grave	4	0,4
DPP^**^	1	0,1
Eclâmpsia	2	0,2
Hipertensão arterial	202	24,3
Infecções	113	14,1
MFF^***^	377	45,2
Placenta prévia	0	0
Outras	36	4,3

* CIUR - Crescimento Intrauterino Restrito;

** DPP - Descolamento prematuro de placenta;

*** MFF - Malformação Fetal.

A combinação dos dados por incorporação está representada no Quadro 1 pelo *joint display,* quadro que os integra e agrupa por meio visual. A exibição contém a primeira coluna relacionada aos pontos de execução do componente qualificação da atenção descritos no projeto Apice On, e a segunda coluna consiste na organização das narrativas mais representativas dos participantes (domínio qualitativo). Já a terceira coluna apresenta alguns indicadores (domínio quantitativo) e, por fim, a quarta especifica as inferências ou *insights* gerados como resultado de complementação, divergências e convergências dos achados^(21)^.

**Quadro 1:** Mixagem dos dados e produção de inferências entre os dados qualitativos e quantitativos

A maternidade de HE e seu cuidado obstétrico segundo o Apice On	Investigação qualitativa	Investigação quantitativa	Inferências, convergências e divergências
A execução do Projeto Apice On Resultados não contemplados, segundo o componente qualificação da atenção •Partos normais de baixo risco assistido por enfermeiras obstétricas	Acho que a gente poderia ter mais esse compartilhamento com a equipe, não só quando eu preciso, só vou compartilhar com você porque eu estou com uma emergência ali. [...] Não é aquele cenário que a gente imaginou, a atuação da enfermeira obstétrica cem por cento [...] realizando o parto de risco habitual. [...] Apesar da peculiaridade do serviço, nós temos gestantes de risco habitual, que dariam perfeitamente pra enfermagem obstétrica acompanhar [...]. (EO-A/1) [...] não tinha um planejamento de como vai ser essa divisão com a equipe médica. Eu acho que se deixasse isso mais amarrado, aos poucos, talvez, as pessoas iriam acostumar com a ideia de a enfermagem obstétrica estar atuando. (EO-A/4) Se a gente tivesse entrado propondo um espaço compartilhado, eu acho que poderia ter sido bem mais aceito do que partos com assistência exclusiva pelo enfermeiro como era proposto no projeto. Pela nossa quantidade baixa e pelo perfil das nossas mulheres que a gente poderia fazer o parto. (EO-A/5) Acho muito complicado quando coisas estão só no papel e simplesmente aparece! E você: “Ué! Quem é? Como é? Como vai ser? Tem limites? Não tem limites?” [...] ninguém sabia como a enfermagem obstétrica estava entrando. Precisava ter sido tão difícil? Não precisava! E quem entra, coitados! Entra também armados, porque também não está sendo bem recebido! (MO-A/9) Eu acho que essa dificuldade foi por causa dessa falta de divulgação, falta de discussão [...] simplesmente chegaram duas enfermeiras obstetras e colocaram lá, entendeu?! E aí todo mundo ficou assim! “E agora?” (MO-A10) Mas, se eu também pactuasse com aquela situação, como iriam ficar os meus residentes? Um parto no dia! Para quem seria o parto? [...] por ser uma maternidade que faz 100 partos/mês e, desses, 50% são cesarianas, temos três turmas de residência médica, [...] e aí eu incorporo o corporativismo, de entender que, dentro dessa maternidade, esse modelo de assistência obstétrica pela enfermeira obstetriz iria tirar espaço do meu residente. [...]. (MO-A/12) E aí chegavam no centro obstétrico na hora da assistência ao trabalho de parto e parto os médicos. Como a maioria não tem experiência com a enfermagem obstétrica, tinham medo da enfermeira atuar. Eu não sei se eles pensam que a gente não tem competência ou se eles não entendem até onde vai. Porque alguns até falam: “Ah! Mas você vai prestar assistência, mas, se complicar, vocês chamam a gente, então não adianta!” [...] Eles têm essa visão de que o enfermeiro tem que estar sempre junto ao médico, exercendo uma coisa que seja meio que “autorizado” pelo médico. (E-GA/14) [...] os residentes até já estão mais acostumados a trabalhar com o enfermeiro obstetra lá fora. Os staffs mais novos também. Mas os staffs antigos não sabiam nem quais são as competências da enfermeira obstétrica [...]. Então era visto muito como se a gente não tivesse a qualificação necessária. Fosse um quebra-galho, sabe?! (E-GUP/18)	- 51,9% dos nascimentos ocorreram por cirurgia cesariana e 48,1% por parto normal. - Dos 43,9% de partos normais de risco habitual, 33,2% correspondiam ao grupo 1 e 31,7% ao grupo 3 da Classificação de Robson. Representam os partos normais potenciais para a evolução fisiológica do trabalho de parto. - 75,7 % das mulheres utilizaram algum tipo de método não farmacológico para alívio da dor. Em linha de base dos meses anteriores à adesão ao projeto, identificou-se 58,3% das parturientes favorecidas com esta boa prática obstétrica. - 1,1 % de partos normais assistidos por enfermeiras obstétricas na sala de parto deste HE.	Os dados quantitativos apresentam um papel suportivo sobre os dados qualitativos. A inserção da enfermeira obstétrica na sala de parto e a assistência a partos normais de risco habitual ampliaram a oferta de métodos não farmacológicos para alívio da dor. Contudo, sua plena atuação nos partos de risco habitual, conforme diretriz do projeto, apresentou uma conjuntura de fatores postos no cenário, que determinaram desvios para atingir o resultado proposto pelo projeto. - O perfil institucional característico de uma unidade referência para gestações de risco materno e fetal determinou o reduzido quantitativo de gestantes classificadas como risco habitual. - Ainda, a deficiência de comunicação entre gestores e corpo assistencial a respeito das diretrizes do projeto afetou o planejamento sobre a assistência obstétrica no parto e as responsabilidades e competências de cada profissional na perspectiva do modelo compartilhado. - O importante questionamento sobre a harmonização entre a missão de um HE e o processo de formação dos residentes médicos.

## DISCUSSÃO

Quanto à inserção das enfermeiras obstétricas, depreende-se a inexistente participação destas na assistência a partos normais de risco habitual na sala de parto deste HE, o que representa um resultado não contemplado segundo o componente qualificação da atenção obstétrica, o qual determinava percentual de 70% para esta meta, de acordo com a matriz de indicadores da atenção obstétrica do projeto Apice On. Vale ressaltar que este dado apresentou movimento de oposição a um estudo avaliativo, que verificou aumento na porcentagem de partos assistidos por enfermeiras obstétricas de 13,7% no estudo Nascer no Brasil para 24,6% na avaliação da Rede Cegonha para a região Sudeste do Brasil^(25)^.

Conforme evidenciado, a presença das enfermeiras obstétricas em sala de parto contribuiu para o incremento do uso de métodos não farmacológicos para alívio da dor, na comparação com dados anteriores à implantação do projeto. Nesse sentido, ainda que seja uma maternidade com perfil de alto risco materno-fetal, valoriza-se a adoção da estratégia de assistência multiprofissional, com a presença destas especialistas, pois são capazes de favorecer a progressão fisiológica do trabalho de parto, a implantação das boas práticas obstétricas, a redução das intervenções durante o trabalho de parto e as taxas de cirurgias cesarianas. Ademais, estabelecem o cuidado relacional, por meio de atitudes de disponibilidade, empatia, acolhimento e sensibilidade^(26)^, que melhoram a satisfação materna quanto à vivencia do parto normal^(6,13-15)^.

Entretanto, mesmo diante dessas evidências, a pesquisa apontou entraves para a atuação destas profissionais, os quais se estruturam em três eixos centrais correlacionados. O primeiro deles fundamenta-se no perfil assistencial de uma maternidade com complexidade materna e fetal, com subsequente reduzido quantitativo de atendimento a gestantes classificadas como risco habitual, que se adequam à legislação e aos critérios para acompanhamento do trabalho de parto pelas enfermeiras obstétricas^(6)^. Essa característica, de certa forma, justificava a não atuação destas especialistas na sala de parto.

No campo da saúde da mulher, os HE são considerados as principais estruturas científicas de transmissão de conhecimentos, referência para gestações de alto risco, o que imputa o pressuposto biomédico de que o corpo feminino necessita de controle e intervenção para a obtenção de resultados biológicos favoráveis^(3,4,27)^. Tais particularidades influenciam as elevadas taxas de procedimentos invasivos, cirurgias cesarianas e baixa incorporação de enfermeiras obstétricas nestas instituições participantes do projeto Apice On^(16,17)^.

Adicionalmente a esse perfil institucional, identificou-se, como segundo fator marcante neste processo de transição organizacional, a fragilidade de comunicação entre gestores e equipe assistencial sobre as diretrizes do projeto e a proposta de um modelo colaborativo de assistência obstétrica por ele suscitado. Na visão de alguns participantes, o fluxo descendente de comunicação organizacional e de diretivas políticas gerou uma deturpação ao longo dessa linha de comunicação devido às interpretações individuais, as quais são afetadas por diferenças culturais, experiências, interesses e expectativas próprios^(28)^.

Tais aspectos, cumpre ressaltar, podem ter induzido um processo de seleção, rejeição, aceitação e interpretação da mensagem e afetado sobremaneira a consciência dos participantes sobre a execução do Projeto Apice On. Isto porque esta é considerada uma importante propriedade da experiência de transição e adquirida a partir do conhecimento e do reconhecimento desse processo, que se reflete no grau de congruência entre o que se sabe e as respostas envolvidas. Em suma, para estar em transição, deve-se ter alguma consciência das mudanças vigentes^(20)^.

Por conseguinte, o nível de engajamento nesta transição organizacional, aquele compreendido pelo grau de interesse demonstrado por uma pessoa no envolvimento no processo, mostrou-se deficiente. Logo, correlaciona-se que o nível de conscientização, alcançado por meio da comunicação adequada, influenciou o nível de engajamento dos profissionais no desenvolvimento desta iniciativa ministerial^(20)^. Nessa perspectiva, evidenciou-se um hiato entre os campos político e prático; uma limitação para a articulação de compromissos e atitudes pertinentes aos gestores, para que convertessem os elementos teóricos presentes no projeto Apice On em ações institucionais internas rumo à modificação para um novo modelo de atenção obstétrica neste HE^(21)^.

Percebe-se, assim, a importância não somente de qualificação contínua dos gestores para o enfrentamento de problemas e desafios ao longo do processo de gestão em saúde, mas, também, no fomento de estratégias para a gestão compartilhada e espaços de escuta^(10)^. De modo semelhante, estudo retratou as dificuldades da gestão para envolvimento e inserção da equipe no desenvolvimento do projeto Apice On, bem como entraves para a capilarização da informação necessária para a compreensão de seus significados, metas e competências a serem mobilizadas pela equipe assistencial^(18)^.

Importante sublinhar também o déficit de comunicação sobre esta proposição ministerial, apontado, principalmente, pela equipe de obstetras, como elemento amplificador da atitude de resistência destes quanto à inserção de enfermeiras obstétricas na sala de parto. Neste processo de transição de modelos de atenção, é frequente haver preocupação por parte dos médicos sobre a experiência e capacidade de atuação das enfermeiras obstétricas e dúvidas sobre a definição de responsabilidades, pois entendem ser de competência técnica exclusiva dos médicos a condução do parto. Já as enfermeiras obstétricas consideram-se responsáveis pelos cuidados às mulheres e discordam de que suas ações devem estar pautadas no campo burocrático ou da submissão^(7,29-31)^.

Nesse ambiente caracterizado por hierarquia, divergências de opiniões e oposição dos modelos de assistência, o tecnocrático e o humanístico, elas enfrentam a tradicional cultura-médico-centrada e lutam pela conquista da autonomia e reconhecimento de suas competências. Nesse sentido, as condutas profissionais demonstram falta de alinhamento, sobretudo da equipe médica, na implantação de práticas colaborativas e de um cuidado obstétrico amparado em evidências científicas^(1,8,27,32,33)^.

Adiciona-se o fato de que, para a teoria das transições, este contexto de trabalho permeado por significados pessoais, culturais e socioeconômicos de cada indivíduo ou categoria pode inibir uma transição organizacional saudável. Ademais, o nível de preparação prévia, de conhecimentos e expectativas pessoais pode interferir no entendimento dos novos papéis exercidos por médicos obstetras e enfermeiras obstétricas em relação às funções a serem por eles desempenhadas na proposta deste novo modelo de atenção obstétrica neste HE^(20)^.

Por conseguinte, o último eixo determinante identificado neste estudo para a não atuação das enfermeiras obstétricas foi o Programa de Residência Médica de Ginecologia e Obstetrícia, com importante peso institucional. O reduzido quantitativo de partos normais de risco habitual, somado a uma possibilidade de divisão destes com o grupo de enfermeiras obstétricas, potencializou discussões e embates. Trata-se de uma dinâmica conflituosa, que dificulta a formulação de sugestões e propostas de ambos os lados para avançar no que realmente determina a estratégia ministerial^(21)^.

Contextualizam-se essas relações sob a compreensão de tais unidades como modelos tradicionais de ensino, nas quais o corpo feminino torna-se objeto de treinamento, através do uso repetitivo e inadequado de técnicas e procedimentos não alinhados às evidências. Por fim, estabelece-se uma relação de que a qualidade da aprendizagem está diretamente relacionada à quantidade de procedimentos assistidos pelos alunos^(4,34)^.

Assim sendo, esta dinâmica de assistência que valoriza a atuação uniprofissional caminha na contramão da Educação Interprofissional em Saúde (EIP), movimento em prol do aperfeiçoamento de habilidades individuais e coletivas e do fortalecimento da comunicação e da atuação em equipe. Tais medidas minimizam a fragmentação do cuidado, aumentam o respeito mútuo entre os profissionais e, por consequência, reduzem comportamentos negativos dos alunos em relação a outras categorias^(35,36)^.

Portanto, a EIP é reconhecida como uma importante estratégia a ser adotada nos HE integrantes do Projeto Apice On, a fim de contemplar o resultado esperado no campo da qualificação do ensino/formação: a formalização do uso de estratégias educacionais que enriqueçam o aprendizado colaborativo entre estudantes e a articulação entre atenção, ensino e trabalho integrado entre equipes multiprofissionais^(10,18)^. Essas diretrizes potencializam a formação adequada de novos profissionais, “forjados na *expertise* das boas práticas obstétricas”, que se constituem como engrenagens para a reconstrução do modelo de atenção ao parto^(34)^.

Nota-se, portanto, que o enfrentamento da enfermeira obstétrica nesse cotidiano assistencial do centro obstétrico, onde a preferência para a condução dos partos é do obstetra, apresenta-se no campo da atenção, por uma deficiente integração entre médicos obstetras, enfermeiras obstétricas, residentes médicos e de enfermagem, e no campo da formação, por uma disputa e segregação durante atuação e aprendizagem em campo prático. Acredita-se que a convivência entre os residentes médicos e de enfermagem em sala de parto oportunize uma valorosa troca interprofissional de conhecimentos e saberes, capaz de ampliar os processos de formação. Há, ainda, a possibilidade de a enfermagem obstétrica demonstrar as práticas humanizadoras que qualificam a assistência materna, e os residentes por sua vez, introduzirem novas perspectivas de cuidado obstétrico ao longo do próprio processo de formação e, com isso, apresentarem menor resistência à tendência de mudança de paradigma no campo do parto e nascimento^(21)^.

Necessário sublinhar que avançar em direção ao modelo colaborativo em obstetrícia, aquele que implementa a participação de enfermeiras obstétricas e obstetrizes na assistência ao trabalho de parto e parto de risco habitual, com acionamento, suporte e condução dos casos com complicações e/ou distocias identificadas por esta profissional, pela equipe de médicos obstetras e nas gestações de alto risco^(14)^, requer uma estreita colaboração entre estes profissionais especializados. Para tanto, são esperadas atitudes de diálogo, confiança e respeito na execução articulada das ações, para que o processo de trabalho em saúde seja elaborado com objetivos e metas comuns, sob uma concepção ampliada do processo saúde-doença. Modelos que garantem mudanças organizacionais em prol da qualidade da atenção à saúde e a democratização do contexto de trabalho são imprescindíveis^(35,37,38)^.

Considera-se, decorridos cinco anos da finalização do Projeto Apice On nesta maternidade de HE, ter sido alcançado um período de equilíbrio nas relações, com amenização dos conflitos entre os obstetras e as enfermeiras obstétricas. Sob a perspectiva da teoria de Meleis, o tempo é uma propriedade do processo de transição, com início marcado por um ponto de partida identificável, neste caso a adesão à proposta ministerial, que se estendeu desde os primeiros sinais de antecipação, percepção ou demonstração de mudança. Em seguida, houve um período de instabilidade, confusão e angústia, sobretudo no que se refere a sentimentos de frustração e impotência vivenciados pelas enfermeiras obstétricas, resultantes da reconstrução de seus papéis neste cenário. A finalização saudável deste processo de transição organizacional foi marcada por um novo começo ou período de estabilidade, decorrente do encerramento do projeto político. Neste momento, os profissionais obtiveram domínio de papéis e padrões de comportamento, necessários para gerenciar situações nesta nova configuração relacional, a qual consolida a presença das enfermeiras obstétricas na sala de parto^(20,21)^.

Assim, ao longo desse processo de reconhecimento sobre as múltiplas adversidades para alcance do propósito do projeto Apice On quanto à assistência a partos normais de risco habitual pelas enfermeiras obstétricas, acabou-se por influenciar reflexões individuais e coletivas sobre o modelo colaborativo ideal a ser instituído nesta maternidade de HE. A incorporação de um modelo colaborativo de atenção obstétrica, embora reconhecido em muitas instituições como satisfatoriamente executável, pode não ser a melhor proposta para outras. Logo, com a experiência vivenciada por este HE, recomenda-se caminhar na construção de modelos de cuidado adequados aos cenários e ao perfil obstétrico, sempre considerando a perspectiva interprofissional do cuidado obstétrico^(21)^.

### Limitações do estudo

Destaca-se a impossibilidade de abranger a percepção de outros atores da equipe multidisciplinar que podem interferir na atuação das enfermeiras obstétricas. O registro manual de informações no livro de indicadores também representa uma limitação, dada a possibilidade de erros de preenchimento. Adicionalmente, aponta-se a inviabilidade de reproduzir com fidedignidade estes resultados para os demais 97 HE participantes do projeto Apice On, pois, conforme mencionado, há peculiaridades relacionadas ao contexto institucional investigado.

### Contribuições para as áreas da Enfermagem, Saúde ou Políticas Públicas

O conhecimento, a descrição e a análise da inserção de enfermeiras obstétricas em uma conjuntura de transição institucional do modelo de assistência obstétrica em HE permitem identificar fatores que afetam o exercício e a autonomia desta profissional durante a práxis de cuidado na assistência a partos normais de risco habitual. Desse modo, são sugeridas importantes estratégias e articulações nas esferas relacional, gerencial e institucional, capazes de viabilizar e ampliar a incorporação destas especialistas, com formação pautada em evidências científicas e preceitos humanísticos, em equipes multidisciplinares. Espera-se, com isso, qualificar a assistência perinatal e, principalmente, tornar tais cenários responsáveis pela formação de atitudes e práticas colaborativas dos futuros profissionais.

## CONSIDERAÇÕES FINAIS

Este estudo descreveu um processo de transição organizacional fomentado pela participação de um HE no projeto Apice On, e a consequente inclusão de enfermeiras obstétricas em sala de parto. Trata-se de uma iniciativa inédita, dada a inexistência de tais especialistas, até então, de modo permanente neste setor.

Válido destacar, contudo, que importantes elementos afetaram a não oficialização de um protocolo institucional que validasse as atribuições legais destas profissionais para o acompanhamento de partos normais de risco habitual. São eles: o perfil de assistência materno-infantil; a deficiente comunicação entre gestores e equipe de plantonistas, considerada um importante recurso gerencial para engajamento e envolvimento de todos os funcionários em prol da política institucional; o desconhecimento dos obstetras sobre as competências e responsabilidades de cada profissional em um modelo compartilhado de assistência obstétrica; e a missão institucional de formação de futuros profissionais, através dos Programas de Residência.

As condições que interferiram na qualidade desta experiência de transição para os sujeitos, como os significados, as expectativas e o nível de conhecimento, conduziram a uma nova configuração assistencial entre enfermeira obstétrica e obstetra na sala de parto, a qual se mantém em equilíbrio até os dias atuais. A equipe passou a compreender as características desta convivência e o escopo dos papéis, pois as enfermeiras obstétricas assumiram caráter coadjuvante nessa dinâmica relacional com o médico obstetra sobre a condução dos partos normais sem distocia, o que culminou na insuficiência de seu papel.

Assim, ao compreender o processo de inserção da enfermeira obstétrica em sala de parto a partir do Projeto Apice On em hospital de ensino, expõe-se a íntima dependência entre os processos de transição organizacional e os espectros que perpassam pelo campo das comunicações, das relações interpessoais e concepções do profissional sobre parto e nascimento. Evidencia-se ainda o tempo necessário para a definição de novos papéis a serem incorporados por obstetras e enfermeiras obstétricas rumo à implantação de novos modelos de assistência obstétrica.

Nesse contexto transicional, assegura-se que o valor da presença e atuação desta profissional está além do reconhecimento da fisiologia do parto para garantir a implementação de práticas recomendáveis, pois também inclui proporcionar cuidado técnico especializado e relacional. Portanto, incentiva-se a discussão, bem como a ampliação, de modelos de cuidados que garantam a inserção das enfermeiras obstétricas em contexto multidisciplinar como pauta permanente nas agendas políticas em saúde da mulher, com abrangência temporal e territorial. Trata-se de uma medida fundamental, por serem profissionais de saúde consideradas fundamentais na prestação de cuidado seguro e qualificado, especialmente em um cenário altamente especializado e de alta complexidade.

## Data Availability

Os dados de pesquisa só estão disponíveis mediante solicitação.
